# miR-483-5p associates with obesity and insulin resistance and independently associates with new onset diabetes mellitus and cardiovascular disease

**DOI:** 10.1371/journal.pone.0206974

**Published:** 2018-11-08

**Authors:** Widet Gallo, Jonathan Lou S. Esguerra, Lena Eliasson, Olle Melander

**Affiliations:** 1 Hypertension and Cardiovascular Disease, Department of Clinical Sciences-Malmö, Lund University, Malmö, Sweden; 2 Lund University Diabetes Centre, Skåne University Hospital, Malmö, Sweden; 3 Islet Cell Exocytosis, Department of Clinical Sciences-Malmö, Lund University, Malmö, Sweden; German Diabetes Center, Leibniz Center for Diabetes Research at Heinrich Heine University Düsseldorf, GERMANY

## Abstract

Our aim was to identify serum microRNAs (miRNAs) in healthy humans which associate with future onset of both diabetes mellitus and cardiovascular disease. We performed global profiling of 753 mature human miRNAs in serum of 12 pilot subjects followed by measurement of 47 consistently expressed miRNAs in fasting serum of 553 healthy subjects from the baseline exam (1991–1994) of the population based Malmö Diet and Cancer Study Cardiovascular Cohort (MDC-CC), of whom 140 developed diabetes, and 169 cardiovascular diseases during follow-up. We used multivariate logistic regression to test individual miRNAs for association with incident diabetes and cardiovascular disease as compared to control subjects (n = 259). After Bonferroni correction and adjustment for age and sex, each SD increment of log-transformed miR-483-5p was significantly associated with both incident diabetes (OR = 1.48; 95% CI 1.18–1.84, P = 0.001) and cardiovascular disease (OR = 1.40; 95% CI 1.15, 1.72, P = 0.001). In cross sectional analysis, miR-483-5p was correlated with BMI (r = 0.162, P = 0.0001), fasting insulin (r = 0.156, P = 0.0002), HDL (r = -0.099, P = 0.02) and triglycerides (r = 0.11, P = 0.01). Adjustment for these metabolic risk factors, as well as traditional risk factors attenuated the miR-483-5p association with incident diabetes (OR = 1.28 95% CI 1.00–1.64, P = 0.049) whereas its association with incident cardiovascular disease remained virtually unchanged (OR = 1.46 95% CI, 1.18–1.81, P = 0.0005). In conclusion, miR-483-5p associates with both diabetes and cardiovascular disease. The association with diabetes seems partly mediated by obesity and insulin resistance, whereas the association with incident cardiovascular disease is independent of these metabolic factors and traditional cardiovascular disease risk factors.

## Introduction

Type-2 diabetes mellitus (diabetes) is a metabolic disease characterized by chronic hyperglycemia resulting from defects in insulin secretion and/or insulin resistance [1, 2]. Patients with diabetes are at increased risk of cardiovascular disease. Additional risk factors for cardiovascular disease are obesity, physical inactivity, alcohol and smoking [3].

MicroRNAs (miRNAs) are endogenous 17–23 nucleotide-long, non-coding RNAs involved in post-transcriptional regulation of mRNA by direct interaction usually at the 3’-untranslated region (UTR) of target mRNA/s resulting in mRNA degradation and/or translational repression [4]. MicroRNAs have been found to stably circulate in the blood, either contained in exosome-like vesicles or associated with protein complexes, and have recently been shown to mediate crosstalk between different cell types [5]. Dysregulated expression of miRNAs has clearly been demonstrated in relevant tissues of diabetes [6, 7] and cardiovascular disease [8], and their emerging roles in disease pathophysiology [9] and potential as disease blood biomarkers has gained ground in recent years [5].

Numerous studies have been performed to identify circulating miRNAs, which are altered in overt diabetes [10–13] and heart diseases [14–16]. However, studies on large and well-powered population-based cohorts linking miRNAs to risk of later development of diabetes and cardiovascular disease are rare. In one prospective population-based cohort with pooled samples for each end-point, Zampetaki *et al*. has shown that plasma levels of mir-126 are reduced at baseline in normoglycemic subjects who later developed diabetes [17]. A follow up study in the same cohort showed a positive association between mir-126 plasma levels and future myocardial infarction [18]. MiRNA-126 was also shown to be predictive for diabetes by Zhang and colleagues [19].

Meanwhile, targeted screening for cardiac miR-21 and miR-150 in plasma showed that reduced levels of the miRNAs associated with atrial fibrillation [20]. Finally, Willeit *et al*. showed that circulating mir-122 levels are associated with development of T2D and components of the metabolic syndrome [21]. Despite all these efforts, the need of powered studies is necessary for more reliable prediction of the onset of diabetes and cardiovascular disease. To the best of our knowledge, no study has investigated large panels of miRNA in non-pooled samples in relation to incident diabetes and cardiovascular disease.

Here we aimed to determine whether serum levels of specific miRNAs are differentially-expressed at baseline in healthy subjects who subsequently developed diabetes and/or cardiovascular disease, in a population-based cohort (n = 5400). We initially determined consistently expressed serum miRNAs in 12 individuals by qPCR-based global profiling of 753 miRNAs, of which 47 miRNAs and internal human U6 snRNA control, were subsequently measured in 553 healthy subjects and levels were compared between those who did and did not develop diabetes or cardiovascular disease later in life.

## Materials and methods

### Subjects and endpoints

The Malmö Diet and Cancer study (MDC) is a population-based, prospective cohort of 28 449 individuals examined between 1991 and 1996 [22]. From this cohort a random sample, examined between November 1991 and February 1994 (n = 6103) were included in the MDC cardiovascular cohort (MDC-CC), with the primary aim to study the epidemiology of carotid artery disease [23]. From all 6103 subjects, 5400 gave fasting blood samples. The samples analyzed in the pilot (n = 12) and the study (n = 553) came from the fasting cohort. At baseline (1991–96) participants underwent a medical history, physical examination and laboratory assessment. Diabetes Mellitus was defined as either self-report of a physician diagnosis, use of diabetes medication or fasting venous whole blood glucose greater than 6.0 mmol/L (109 mg/dL). Levels of HDL-C, total cholesterol, triglycerides and insulin were measured according to standard procedures at the Department of Clinical Chemistry, University Hospital of Malmö. The levels of LDL-C were calculated according to the Friedewald formula. All serum samples were obtained after overnight fasting and samples were drawn between 7.30 am and 9.00 am.

New-onset diabetes cases occurring during a mean follow-up time of 16 years were retrieved through record linkage using the Swedish personal identification number with six different national and regional diabetes registers: Individuals could be registered as having a diagnosis of diabetes in the nationwide Swedish National Diabetes Register [24] or in the regional Diabetes 2000 register of the Scania region of which Malmö is the largest city [25] or in the Swedish National Patient register, which is a principal source of data for numerous research projects that covers more than 99% of all somatic and psychiatric hospital discharges and Swedish Hospital-based outpatient care [26]; or they could be classified as diabetes cases if they had diabetes as a cause of death in the Swedish Cause of Death Register, which comprises all deaths among Swedish residents occurring in Sweden or abroad [27] or if they had been prescribed anti-diabetic medication as registered in the Swedish Prescribed Drug Register [28]; or if they had at least two HbA1c recordings of ≥ 6.0% using the Swedish Mono-S standardization system, corresponding to ≥ 7.0% according to the US National Glycohemoglobin Standardization Program (NGSP), in the Malmö HbA1c register, which analyzed and catalogued all HbA1c samples at the Department of Clinical Chemistry taken in institutional and non-institutional care in the greater Malmö area from 1988 onwards.

New-onset cardiovascular disease cases that occurred during a follow-up time of 16 years were identified through record linkage of the 10-digit personal identification number of each Swedish citizen with three registries: Swedish National Patient register [26]; the Swedish Cause of Death Register [27] and the Stroke in Malmö register [29]. Cardiovascular disease events were defined as coronary events or fatal or non-fatal stroke, whichever came first. Coronary events were defined as fatal or non-fatal myocardial infarction or death due to ischemic heart disease. Myocardial infarction was defined as per the *International Classification of Diseases* 9^th^ and 10^th^ Revisions (ICD9 and ICD10) codes 410 and I21, respectively. Death due to ischemic heart disease was defined based on codes 412 and 414 (ICD9) or I22-I23 and I25 (ICD10). Fatal and non-fatal stroke was defined using codes 430, 431, 434 and 436 (ICD9) and I60, I61, I63, and I64 (ICD10).

From the MDC-CC we first randomly selected 4 subjects free from prevalent and incident diabetes and cardiovascular disease, 4 subjects who developed diabetes and 4 subjects who developed cardiovascular disease during follow-up and profiled individual serum samples from these 12 subjects for a panel of 753 human miRNAs. Thereafter we measured 47 miRNAs, which were consistently detected in all 12 pilot samples in another 553 unique subjects. These consisted of 259 control subjects (randomly selected from the MDC-CC among subjects free from both prevalent and incident diabetes and cardiovascular disease), 140 incident cases of diabetes (randomly selected from the MDC-CC among subjects free from diabetes at baseline who developed diabetes during follow-up) and 169 incident cases of cardiovascular disease (randomly selected from the MDC-CC among subjects free from cardiovascular disease at baseline who developed cardiovascular disease during follow-up). We had missing data on insulin levels for 5 subjects, but we decided to not exclude them as insulin is not a well-established risk factor for diabetes.

**Fig 1:**
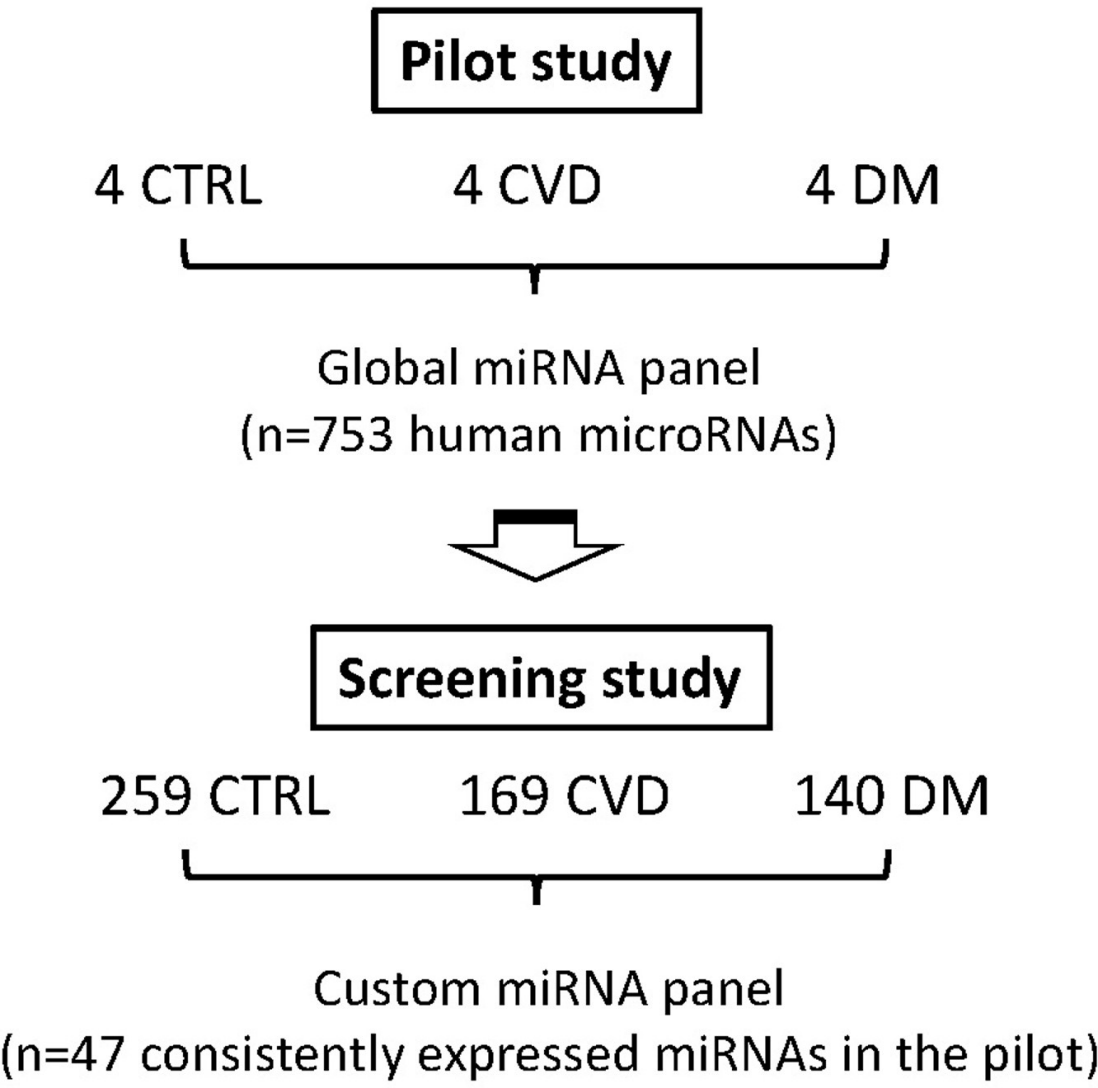
Study design. A pilot study was performed using a qPCR-based panel in which 753 human miRNAs were measured from 12 individual fasting serum samples from CTRL (control), CVD (cardiovascular disease) and DM (diabetes mellitus) groups. For the screening study, 47 consistently expressed miRNAs determined in the pilot study were used in a custom miRNA panel to screen 259 CTRL, 169 CVD and 140 DM samples.

The study was approved by the Regional Board of Ethics in Lund, Sweden. A flow chart showing the study design is shown in Fig 1.

### RNA isolation

Total RNA from human serum was extracted using the miRNeasy 96 Total RNA isolation kit (Qiagen) using a modified protocol. Briefly, serum samples were thawed in ice and 250 μL was centrifuged in 1.5 mL nuclease-free microcentrifuge tubes for 5 min at 1000g at 4°C to remove debris. 200 μL of the serum samples were then transferred to new tubes, and 800 μL of Qiazol with 1.25 μL of carrier RNA, MS2 RNA (0.8 ug/uL, Roche Cat. #10165948001) was then thoroughly mixed with the serum. To extract the total RNA, 200 μL of chloroform (Sigma Aldrich, Cat. #C2432) was added and the mixture was further processed according to the manufacturer’s recommendations. The total RNA was eluted with 50 μL RNase-free water and stored at −80°C.

### cDNA synthesis and qPCR

To determine the expression of miRNA stably-expressed in the serum, we performed a pilot experiment on 12 serum samples. We performed cDNA synthesis with pre-amplification, first by reverse transcription using the Megaplex RT Primers, Human Pool Set v3.0 (Cat # 4444745) in Taqman microRNA Reverse Transcription Kit (Cat # 4366596), followed by Megaplex PreAmp Primers, Human Pool Set v3.0 (Cat # 4444748) in Taqman pre-amp Master Mix (Cat# 4391128) according to manufacturer’s recommendations.

We then performed qPCR using Taqman Low-Density Array (TLDA) cards pre-loaded with Taqman primer/probe specific for 753 mature human miRNAs, small RNAs U6 snRNA, RNU44, RNU48 and negative control miRNAs from *A*. *thaliana* (ath-miR-159a) and *D*. *melanogaster* (dme-miR-7) (TaqMan Array Human MicroRNA A+B Cards Set v3.0 (Cat# 4444913) in ViiA7 Real-Time PCR system (Thermo-Fisher Scientific). For the qPCR of 47 selected miRNAs and U6 snRNA, we designed custom TLDA cards which can accommodate up to 8 samples per card. Reverse transcription with pre-amplification and qPCR were performed using customized primers and primer/probe mixes specific only for the selected miRNAs.

QPCR data is deposited in the ArrayExpress database at EMBL-EBI (www.ebi.ac.uk/arrayexpress) under accession number E-MTAB-7246.

### Data analysis and normalization for relative quantification of miRNA

For the pilot run (N = 12 samples), included in the TLDA cards are negative miRNA assay controls: 1) specific for *A*. *thaliana*, ath-miR-159a and 2) for *D*. *melanogaster* dme-miR-7, which were all “Undetermined” in all sample wells, confirming no plant/insect miRNA contamination and unspecific amplifications in our serum samples.

To determine the miRNA set to be used for screening of the larger cohort (N = 553), we had to set a strict criterion in which only the microRNAs expressed in all our 12 pilot samples were included. We therefore excluded miRNAs with “Undetermined” in any sample and set the detection limit to Ct ≤ 37. We determined that 48 miRNA assays out of 753 were expressed in all twelve samples. However due to plate design technical limitation, we had to choose only 47 miRNAs to screen the N = 553 cohort. U6 snRNA was included by default in the custom TLDA plates but was not used as endogenous control in this study.

To identify endogenous controls, we evaluated the 15 miRNA assays expressed in all 553 samples of which, hsa-miR-17 and hsa-miR-106a were found to be the most stably expressed miRNAs according to the GeNORM procedure [30] (S1 Fig). Notably, hsa-miR-17 was previously used as normalizer in a miRNA profiling study of 424 patients in relation to circulating cardio-enriched miRNAs in myocardial infarction [16]. Here, we used the geometric means of hsa-miR-17 and hsa-miR-106a as endogenous controls in the relative quantification using the 2^−ΔΔCt^ method.

### Statistics

For the 47 miRNAs consistently expressed in the 12 pilot samples, we used age and sex adjusted logistic regression to calculate odds ratios (OR) per one standard deviation increment of log-transformed expression levels to incident diabetes versus control status and to incident cardiovascular disease vs control status.

Power calculations show that with the numbers of incident diabetes cases (n = 140) and controls (n = 250), we had a power of 0.81 to detect a b-coefficient of 0.3 (i.e., an effect estimate smaller than the one actually found) on the standardized scale of miR-483-5p in relation to incident diabetes (versus controls) with alpha = 0.05. For incident CVD cases (n = 169) and controls (n = 259), we had a power of 0.86 to detect a b-coefficient of 0.3 (i.e., an effect estimate smaller than the one actually found) on the standardized scale of miR-483-5p in relation to incident CVD (versus controls) with alpha = 0.05.

For miRNAs showing significant association after Bonferroni correction (P<0.05 for 47 tested miRNAs, i.e. P<0.00104), we then performed cross sectional Pearson correlation analysis between cardiometabolic risk factors at baseline in all 553 subjects and the miRNA in question. Subsequently, we additionally adjusted for established diabetes risk factors and cardiometabolic risk factors with significant correlation with the miRNA in question in the analysis of incident diabetes (age, sex, body mass index, high density lipoprotein-cholesterol, fasting insulin and triglyceride concentrations) and for established cardiovascular disease risk factors and cardiometabolic risk factors with significant correlation with the miRNA in question in the analysis of incident cardiovascular disease (age, sex, systolic blood pressure, anti-hypertensive treatment, current smoker, low density lipoprotein-cholesterol, high density lipoprotein-cholesterol, diabetes mellitus, body mass index, fasting insulin and triglyceride concentrations).

Finally, to test whether the association between such miRNA and incident diabetes and cardiovascular disease, respectively, was linear or not, we related ascending quartile of miRNA to incident diabetes and cardiovascular disease, with the lowest miRNA quartile as the reference.

## Results

Out of 753 miRNAs measured in the pilot study, 47 consistently expressed were further analyzed in the main study (Fig 1). The baseline characteristics of the three study groups are shown in Table 1, while S2 Table shows the subcohort in comparison to the background cohort.

**Table 1:** Baseline clinical characteristics of case-control.

	Incident Cardiovascular disease cases	Incident Diabetes cases	Controls
**Sample size (N)**	169	140	259
**Age (Years)** ^α^	60.3 (5.2)	57.3 (6.0)	59.4 (5.7)
**body-mass-index (kg/m^2^)** ^α^	26.7 (4.3)	28.7 (5.1)	26.5 (4.1)
**Low density lipoprotein (mmol/l)** ^α^	4.4 (0.9)	4.3 (0.9)	4.2 (0.9)
**High density lipoprotein (mmol/l)** ^α^	1.3 (0.3)	1.2 (0.3)	1.3 (0.3)
**Systolic blood pressure (mm Hg)** ^α^	149.8 (17.9)	148.2 (20.3)	145.9 (18.2)
**Triglycerider (mmol/L)** ^α^	1.4 (0.6)	1.6 (0.7)	1.4 (0.7)
**Insulin (μU/mL)** ^α^	n = 167 (2.0 (0.6))	n = 139 (2.3 (0.5))	n = 257 (1.9 (0.5))
**Anti Hypertension Treatment %** ^b^	23.1	27.1	24.7
**Diabetes %** ^b^	13.0	0	3.5
**Current smoker, n (%)**^b^	35.5	30.7	29.3
**Sex (% women)** ^b^	45.6	50.7	52.9

^α^Values for the variables are displayed as mean (SD, Standard Deviation).

^b^Frequency in percent.

The age and gender adjusted odds ratios for incident diabetes and incident cardiovascular disease, expressed per one standard deviation increment of log-transformed value of the miRNA in question, are shown in S1 Table. Notably, miR-483-5p was significantly associated with risk of both incident diabetes and incident cardiovascular disease even after Bonferroni correction (Tables 2 and 3).

**Table 2:** miR-483-5p and incident diabetes.

	Continuous analysis (per SD increment)	P-value	Quartile 1	Quartile 2	Quartile 3	Quartile 4	P for trend
**N / N events** ^α^	390/140		97 / 31	98 / 27	98 / 36	97 / 46	
**OR (95% CI) (age and sex adjusted)**	1.48 (1.18–1.84)	0.001	1.0 (ref)	0.84 (0.45–1.56)	1.24 (0.68–2.25)	2.11 (1.16–3.83)	0.006
OR (95% CI) (fully adjusted)*	1.28 (1.00–1.64)	0.049	1.0 (ref)	0.64 (0.33–1.27)	0.97 (0.51–1.82)	1.40 (0.73–2.68)	0.167

^α^ Values for the variables are displayed as mean (SD, Standard Deviation); 95% confidence interval (CI) for odds ratio (OR).

*Adjusted for Age, Sex, Body Mass Index, High Density Lipoprotein; Insulin and Triglycerides.

**Table 3:** miR-483-5p and incident cardiovascular disease.

	Continuous analysis (per SD increment)	P-value	Quartile 1	Quartile 2	Quartile 3	Quartile 4	P for trend
**N / N events**^a^	428/169		71 / 36	68 / 39	65 / 42	55 / 52	
**OR (95% CI) (age and sex adjusted)**	1.40 (1.14–1.71)	0.001	1.0 (ref)	1.09 (0.62–1.93)	1.26 (0.72–2.21)	1.80 (1.03–3.14)	0.032
OR (95% CI) (fully adjusted)*	1.46 (1.18–1.84)	0.0005	1.0 (ref)	0.95 (0.52–1.72)	1.14 (0.63–2.07)	1.80 (1.00–3.24)	0.037

^α^ Values for the variables are displayed as mean (SD, Standard Deviation); 95% confidence interval (CI) for odds ratio (OR).

*Adjusted for age, sex, systolic blood pressure, Anti-hypertensive treatment, current smoker, Low density lipoprotein, High density lipoprotein, Diabetes Mellitus, triglycerides, body mass index and insulin.

In continuous analysis, after adjustment for age and sex for both diabetes and CVD, Quartile 4 is higher than Quartile 1 with significant trends. The Quartiles 2 and 3 are not significant in relation to the reference, Quartile 1.

In correlation analysis in all 553 subjects, there was significant positive relationship between baseline level of log-transformed miR-483-5p and body mass index, waist circumference, fasting insulin and triglyceride concentrations and significant negative correlation between log transformed miR-483-5p and HDL-cholesterol (Table 4).

**Table 4:** Correlation between miR-483-5p and classical risk factors.

	Age	Sex	BMI	Waist	Insulin	HDL	Triglycerides
**N**	553	553	553	553	548	553	553
**Pearson Coefficient**	0.041	0.013	0.162**	0.135**	0.156**	0.099*	0.110**
**Sig. (2-tailed)**	0.340	0.766	0.0001	0.001	0.0002	0.020	0.010

miR-483-5p correlated positively with (BMI) Body Mass Index, Waist, Insulin, High Density Lipoprotein (HDL) and Triglycerides.

* Correlation coefficients significant at 0.05 level.

** Correlation coefficients significant at 0.01 level.

After additional adjustment for these cardiometabolic risk factors as well as established diabetes and cardiovascular disease risk factors, the association between miR-483-5p and incident diabetes was attenuated, whereas the association between miR-483-5p and incident cardiovascular disease remained significant with similar effect size as in the age and sex adjusted model (Tables 2 and 3). Interestingly miR-126, which in other prospective studies has been shown to be inversely related to incident diabetes [17, 19] and positively related to incident myocardial infarction [18] was not related to neither incident diabetes nor to incident cardiovascular disease in our study after Bonferroni correction (S1 Table). However, we found a positive nominally-significant relationship between mir-126 and incident diabetes (P = 0.01).

## Discussion

Diabetes mellitus is a major risk factor for cardiovascular disease. Given recent studies on the role of circulating miRNAs in cell-to-cell signaling [31, 32], we hypothesize that circulating miRNAs, could either reflect a common sub-clinical condition of cardiometabolic disease or even be part of an organ-to-organ signaling which contributes to development of cardiometabolic disease.

The key finding of our study is that out of 47 miRNAs which we could identify at detectable levels in serum in a pilot study of 12 subjects, serum concentration of one miRNA, i.e. miR-483-5p, significantly associated with later onset of both diabetes and cardiovascular disease. Interestingly, miR-483-5p concentration was cross-sectionally correlated with metabolic risk factors at baseline, such as measures of obesity, insulin resistance and dyslipidemia and when we adjusted for these factors, the association between miR-483-5p and diabetes was attenuated, whereas the association with cardiovascular disease remained unchanged. This suggests that the association between miR-483-5p and diabetes could be mediated by obesity, insulin resistance and dyslipidemia, whereas its association with cardiovascular disease may be explained by other pathways.

Indeed, miR-483-5p is an intronic miRNA shown to be co-expressed with its host gene *IGF-2* (Insulin-like growth factor 2), and demonstrated to putatively target SOCS3 (suppressor of cytokine signaling-3) [33]. Both IGF-2 and SOCS3 have been suggested to play a role in obesity development [34] and in the regulation of insulin resistance [35], allowing us to speculate that the higher levels of miR-483-5p may interplay with IGF-2 and/or SOCS3 and thereby contribute to obesity, insulin resistance and risk of diabetes development.

Interestingly, in patients undergoing cardiac surgery, elevated pre-operative levels of mir-483-5p both in right atrial biopsies and in serum have been shown to predict post-operative atrial fibrillation [36]. This suggests that miR-483-5p is upregulated and secreted in patients with myocardial stress, a scenario resembling that of cardiac natriuretic peptides [37]. One can therefore speculate that, apart from having metabolic effects, miR-483-5p is upregulated in healthy subjects and secreted from the heart in conditions of subclinical cardiac wall stress and therefore predicts development of cardiovascular disease later in life.

Only few prospective studies have investigated the impact of microRNAs on heart disease risk prediction and all were characterized by relatively small samples size. From this point of view, our study has several strengths. The first strength of our study is its initial unbiased quantitative PCR-based, profiling of 753 mature microRNAs in non-pooled serum samples from twelve healthy individuals, of which four later developed diabetes and four later developed cardiovascular disease. Our study is opposed to the Bruneck study in which the initial global screening of dysregulated miRNAs was from pooled plasma samples of diabetes cases (N = 5) *vs* controls (N = 5) [17]. There are also other differences in design between our study and the Bruneck study that may explain differences in results, such as the fact that we exclusively used incident cases of diabetes.

Considering our blind approach, we were very strict in including only the 47 miRNAs consistently expressed in all twelve serum samples in the subsequent screening of the larger cohort of 553 serum samples from healthy humans (the 12 samples used in the pilot are not included in this study). This is the second strength of our study, and to our knowledge is the biggest longitudinal study with 553 healthy humans that has been used to determine whether serum levels of miRNAs are differed in expression at baseline in healthy subjects who later developed diabetes and/or cardiovascular disease, in a population-based cohort. Despite the relatively large size of our study, additional studies are needed on diabetes and especially heart diseases to confirm our results.

A weakness of our study is the strict criterion of including only 47 consistently-expressed miRNAs found in the pilot screening of only 12 serum samples, a low number sample size with only four cases per condition. It is possible that in the pilot screening, we inadvertently excluded circulating miRNAs that were differentially-regulated between cases and controls. Nonetheless, we observed many of the miRNAs we identified that have also been identified in previous studies. For instance, we could detect miR-126 and other correlated miRNAs that have been previously associated with diabetes and cardiovascular disease [17, 18], although we could not replicate previous findings.

A potential explanation on the observed discordance, in addition to differences in study design and statistical power, is in the use of serum in our study. Although the expression of miRNAs in serum and plasma have been shown to strongly correlate [38, 39], previous comparisons showed serum samples to contain higher concentrations of miRNAs than plasma [40]. It has also been shown that the vast majority of miRNAs found in serum are contained within exosomal vesicles [41] which facilitate miRNA trafficking between cells.

The observed upregulation of serum miRNAs in the diseased state in our study supports the hypothesis that these miRNAs could be involved in cell-to-cell communication. However, a more in-depth study is required to ascertain the cellular origins and destination of the miRNAs in question. It should also be mentioned that in our study we have retrieved incident cases of diabetes through record linkage with registries and for that reason we cannot determine the subtype of diabetes. Hence, here we assumed that majority of patients with diabetes has type 2 diabetes since the average age at baseline was greater than 55 years old.

In conclusion, we show that miR-483-5p is associated with obesity and insulin resistance and independently associates with new onset diabetes mellitus and cardiovascular disease. The expression of miR-483-5p in serum is reliable as a non-invasive biomarker for its association with cardiovascular diseases and diabetes and it could easily be used in clinical studies.

## Supporting information

S1 Fig Average expression stability values of remaining control genes. Fifteen miRNA assays expressed in all 553 samples were evaluated to identify endogenous controls. According to GeNORM procedure, hsa-miR-17 and hsa-miR-106a were most stably expressed. The geometric means of hsa-miR-17 and hsa-miR-106a were used as endogenous controls in the relative quantification using the 2−ΔΔCt method.
(TIF)

S1 Table Bonferroni correction for CVD and DM. Adjusted for Age and Sex; miR-483-5p is significantly associated with risk of both incident diabetes and incident cardiovascular disease after Bonferroni correction; n.s: non-significant.
(PDF)

S2 Table Baseline clinical characteristics of subcohort and background cohort. Baseline clinical characteristics of subcohort (553) vs. the background cohort (6094).
(PDF)
