# Supplementary figures and images for: miR-483-5p associates with obesity and insulin resistance and independently associates with new onset diabetes mellitus and cardiovascular disease
Source: PLoS One. 2018 Nov 8;13(11):e0206974. doi: 10.1371/journal.pone.0206974 (PMC6224079; doi:10.1371/journal.pone.0206974)

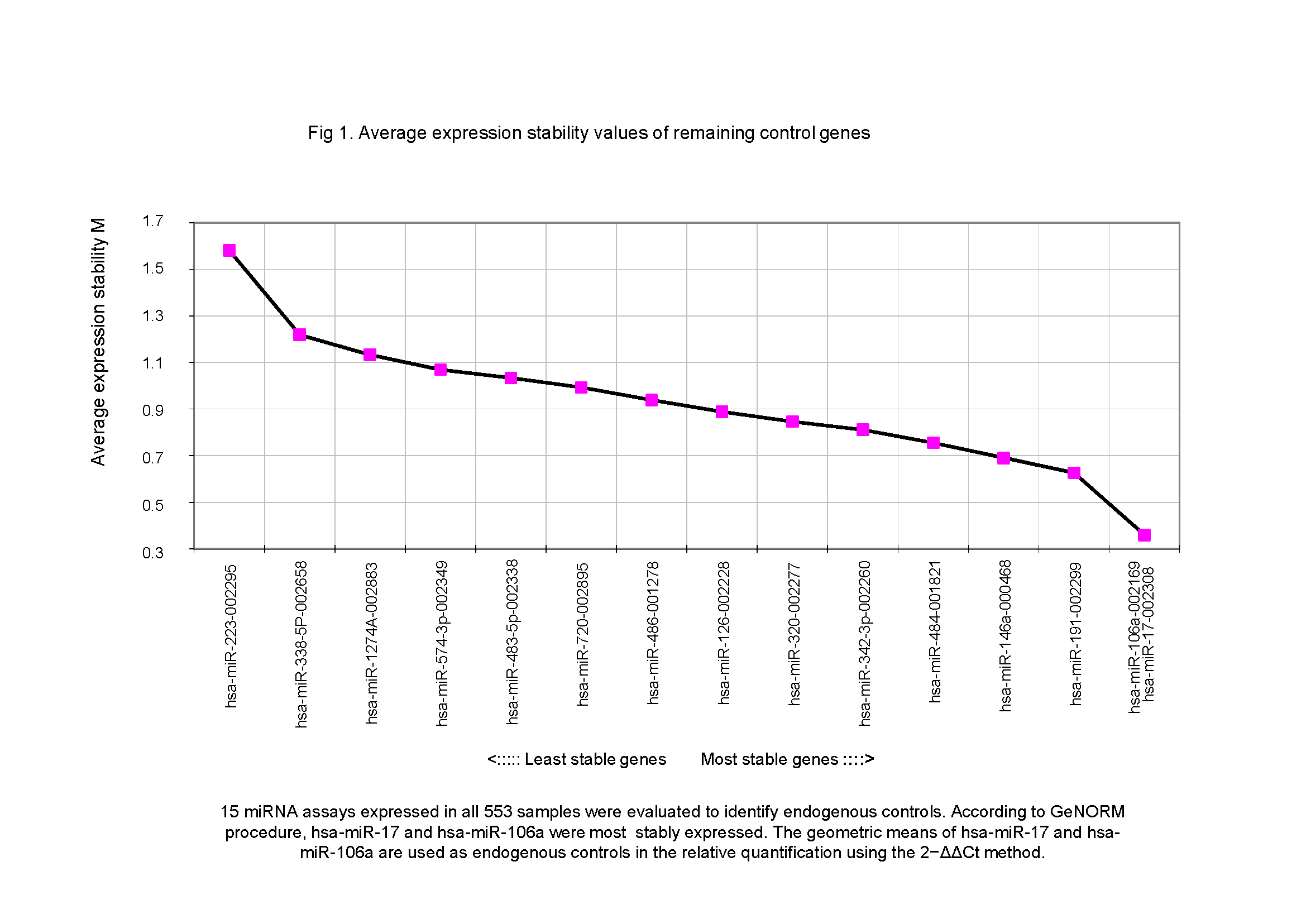

Supplement: S1 Fig — Fifteen miRNA assays expressed in all 553 samples were evaluated to identify endogenous controls. According to GeNORM procedure, hsa-miR-17 and hsa-miR-106a were most stably expressed. The geometric means of hsa-miR-17 and hsa-miR-106a were used as endogenous controls in the relative quantification using the 2−ΔΔCt method. (TIF) [file pone.0206974.s001.tif]
