# Supplementary material for: miR-483-5p associates with obesity and insulin resistance and independently associates with new onset diabetes mellitus and cardiovascular disease
Source: PLoS One. 2018 Nov 8;13(11):e0206974. doi: 10.1371/journal.pone.0206974 (PMC6224079; doi:10.1371/journal.pone.0206974)
Supplement: S1 Table — Adjusted for Age and Sex; miR-483-5p is significantly associated with risk of both incident diabetes and incident cardiovascular disease after Bonferroni correction; n.s: non-significant. (PDF) [file pone.0206974.s002.pdf]

S1 Table. Bonferroni correction for CVD and DM

Cardiovascular, CVD					
**Index**	**MicroRNA**	**Beta**	**95% CI**	**P-**	**After Bonferroni**
1	***miR-483-5p***	***1,4***	***(1.14-1.71)***	***0,001***	***0,048***
2	miR-7b	1,28	(1.05-1.56)	0,013	n.s
3	miR-7d	1,04	(0.86-1.27)	0,648	n.s
4	miR-7e	1,11	(0.92-1.35)	0,262	n.s
5	miR-160	0,93	(0.76-1.14)	0,521	n.s
6	miR-170	1,03	(0.85-1.26)	0,698	n.s
7	miR-210	1,02	(0.84-1.24)	0,791	n.s
8	miR-240	1.1 (	0.91-1.34)	0,311	n.s
9	miR-250	1,01	(0.83-1.23)	0,88	n.s
10	miR-122	1,23	(1.01-1.5)	0,037	n.s
11	miR-126	1,12	(0.92-1.36)	0,251	n.s
12	miR-145	1,12	(0.92-1.36)	0,249	n.s
13	miR-150	1,11	(0.91-1.35)	0,286	n.s
14	miR-186	1,07	(0.88-1.3)	0,49	n.s
15	miR-191	1,14	(0.94-1.39)	0,172	n.s
16	miR-197	1,12	(0.92-1.37)	0,225	n.s
17	miR-222	1,12	(0.92-1.37)	0,223	n.s
18	miR-223	1,03	(0.85-1.25)	0,733	n.s
19	miR-320	1,18	(0.97-1.43)	0,092	n.s
20	miR-331	1,06	(0.87-1.29)	0,51	n.s
21	miR-335	0,96	(0.79-1.17)	0,71	n.s
22	miR-374	1,04	(0.85-1.26)	0,68	n.s
23	miR-484	0,98	(0.8-1.19)	0,85	n.s
24	miR-486	1,08	(0.89-1.31)	0,405	n.s
25	miR-625	1,21	(0.84-1.74)	0,296	n.s
26	miR-720	1,08	(0.89-1.32)	0,4	n.s
27	miR-1243	1,17	(0.9-1.52)	0,232	n.s
28	miR-106a	0,94	(0.78-1.15)	0,606	n.s
29	miR-125a	1,07	(0.88-1.3)	0,443	n.s
30	miR-1274a	1,18	(0.97-1.43)	0,095	n.s
31	miR-1274b	1,24	(1.02-1.51)	0,029	n.s
32	miR-1395p0	1,09	(0.89-1.32)	0,367	n.s
33	miR-1423p0	0,92	(0.76-1.13)	0,463	n.s
34	miR-146a	0,96	(0.78-1.17)	0,695	n.s
35	miR-146b	1,23	(1.01-1.5)	0,033	n.s
36	miR-19b	1,02	(0.84-1.23)	0,839	n.s
37	miR-20a	0,98	(0.81-1.2)	0,912	n.s
38	miR-20b	0,99	(0.81-1.21)	0,966	n.s
39	miR-26a	1,08	(0.89-1.32)	0,388	n.s
40	miR-283p0	0,93	(0.76-1.13)	0,488	n.s
41	miR-30b	1,11	(0.91-1.35)	0,281	n.s
42	miR-30c	1,11	(0.91-1.35)	0,268	n.s
43	miR-3385p0	1,19	(0.98-1.45)	0,074	n.s
44	miR-3423p0	1,18	(0.97-1.44)	0,082	n.s
45	miR-5743p0	1,11	(0.92-1.35)	0,259	n.s
46	miR-92a	1,13	(0.93-1.37)	0,215	n.s
47	miR-	1	(0.83-1.22)	0,951	n.s
48	miR-UssnRNA	1,02	(0.84-1.25)	0,766	n.s

S1 Table. Bonferroni correction for CVD and DM

Diabetes Mellitus, DM					
**Index**	**MicroRNA**	**Beta**	**95%CI**	**P-**	**After Bonferroni**
1	***miR-483-5p***	***1,48***	***(1.18-1.84)***	***0,001***	***0,048***
2	miR-7b	1,22	(0.98-1.49)	0,068	n.s
3	miR-7d	1,09	(0.88-1.34)	0,413	n.s
4	***miR-7e***	***1,45***	***(1.16-1.79)***	***0,001***	***0,048***
5	miR-160	1,15	(0.93-1.41)	0,194	n.s
6	miR-170	0,85	(0.68-1.05)	0,147	n.s
7	miR-210	1,1	(0.89-1.35)	0,373	n.s
8	miR-240	1,22	(0.98-1.5)	0,065	n.s
9	miR-250	-	-	0,975	n.s
10	miR-122	1,25	(1.01-1.54)	0,036	n.s
11	miR-126	1,32	(1.06-1.62)	0,011	n.s
12	miR-145	1,1	(0.89-1.35)	0,357	n.s
13	miR-150	1,24	(1-1.53)	0,045	n.s
14	miR-186	1,21	(0.98-1.49)	0,076	n.s
15	miR-191	1,24	(1-1.53)	0,048	n.s
16	miR-197	1,16	(0.93-1.42)	0,178	n.s
17	miR-222	1,35	(1.09-1.67)	0,005	n.s
18	miR-223	1,1	(0.89-1.34)	0,368	n.s
19	miR-320	1,3	(1.05-1.61)	0,014	n.s
20	miR-331	0,99	(0.79-1.21)	0,9	n.s
21	miR-335	0,98	(0.79-1.2)	0,847	n.s
22	miR-374	1,01	(0.81-1.24)	0,923	n.s
23	miR-484	1,08	(0.88-1.33)	0,48	n.s
24	miR-486	1,2	(0.97-1.48)	0,081	n.s
25	miR-625	1,2	(0.88-1.63)	0,23	n.s
26	miR-720	1,36	(1.09-1.68)	0,005	n.s
27	miR-1243	2,32	(3.16-1.7)	0,995	n.s
28	miR-106a	1,18	(0.93-1.47)	0,164	n.s
29	miR-125a	1,06	(0.86-1.3)	0,575	n.s
30	miR-1274a	1,37	(1.1-1.69)	0,004	n.s
31	miR-1274b	1,33	(1.07-1.64)	0,009	n.s
32	miR-1395p0	1,12	(0.91-1.38)	0,273	n.s
33	miR-1423p0	1,11	(0.9-1.36)	0,325	n.s
34	miR-146a	1,13	(0.9-1.39)	0,278	n.s
35	miR-146b	1,19	(0.96-1.47)	0,102	n.s
36	miR-19b	1,15	(0.93-1.42)	0,182	n.s
37	miR-20a	1,03	(0.83-1.26)	0,807	n.s
38	miR-20b	1,06	(0.85-1.3)	0,59	n.s
39	miR-26a	1,28	(1.03-1.58)	0,025	n.s
40	miR-283p0	0,8	(0.64-0.99)	0,05	n.s
41	miR-30b	1,3	(1.04-1.6)	0,017	n.s
42	miR-30c	1,13	(0.91-1.39)	0,268	n.s
43	miR-3385p0	1,36	(1.09-1.67)	0,005	n.s
44	miR-3423p0	1,31	(1.05-1.61)	0,013	n.s
45	miR-5743p0	1,2	(0.97-1.48)	0,087	n.s
46	miR-92a	1,17	(0.95-1.44)	0,132	n.s
47	miR-	1,06	(0.86-1.31)	0,569	n.s
48	miR-UssnRNA	1,05	(0.85-1.29)	0,628	n.s

Adjusted for Age and Sex; miR-483-5p is significantly associated with risk of both incident diabetes and incident cardiovascular disease after Bonferroni correction; n.s: non-significant
