# Supplementary material for: miR-483-5p associates with obesity and insulin resistance and independently associates with new onset diabetes mellitus and cardiovascular disease
Source: PLoS One. 2018 Nov 8;13(11):e0206974. doi: 10.1371/journal.pone.0206974 (PMC6224079; doi:10.1371/journal.pone.0206974)
Supplement: S2 Table — Baseline clinical characteristics of subcohort (553) vs. the background cohort (6094). (PDF) [file pone.0206974.s003.pdf]

S2.Table Baseline clinical characteristics of subcohort and bakground Cohort

	subcohort	background cohort
	**n= 553**	**n= 6094**
**Age (Years)** [α]	59.2 (5.8)	57.5 (5.9)
**body-mass-index (kg/m²)** [α]	27.1 (4.6)	25.9 (4.0)
**Low density lipoprotein (mmol/l)** [α]	4.3 (0.9)	4.2 (1.0)
**High density lipoprotein (mmol/l)** [α]	1.3 (0.3)	1.4 (0.4)
**Systolic blood pressure (mm Hg)** [α]	147.7 (19)	141.4 (19.1)
**Triglycerider (mmol/L)** [α]	1.5 (0.7)	1.4 (0.8)
**Insulin (µU/mL)** [α]	2 (0.6)	2 (0.6)
**Anti Hypertension Treatment %** [b]	24.2	16.6 (0.4)
**Diabetes %** [b]	6.9	8.8
**Current smoker, n (%)**[b]	32.2	26.5
**Sex (% women)** [b]	51.2	57.8

[α] Values for the variables are displayed as mean (SD, Standard Deviation) or as frequency in percent [b].
